# Staging reinterventions for remodeling of residual aortic dissection: a single-center retrospective study

**DOI:** 10.3389/fcvm.2024.1360830

**Published:** 2024-05-10

**Authors:** Bailang Chen, Kunpeng Huang, Xianmian Zhuang, Zanxin Wang, Minxin Wei

**Affiliations:** ^1^ Department of Cardiovascular Surgery, The University of Hong Kong-Shenzhen Hospital, Shenzhen, Guangdong, China; ^2^ Department of Medicine, Shenzhen University, Shenzhen, Guangdong, China

**Keywords:** staging reintervention, residual aortic dissection, aortic remodeling, entry tear, endovascular aortic repair

## Abstract

**Objective:**

Inadequate remodeling of residual aortic dissection (RAD) following repair of Stanford A or B aortic dissections has been identified as a significant predictor of patient mortality. This study evaluates the short- to mid-term outcomes of staged reinterventions for RAD at a single center with prospective follow-up.

**Methods:**

Data were retrospectively collected from patients with RAD who underwent staged reinterventions or received none-surgery treatment in the Cardiovascular Surgery Department of our hospital between July 2019 and December 2021. The cohort included 54 patients with residual distal aortic dissection post-primary surgery, comprising 28 who underwent open surgery and 26 who received thoracic endovascular aortic repair (TEVAR). Patients were divided into two groups: those who underwent staged stent interventions for distal dissection [staged reintervention (SR) group] and those who did not undergo surgery (non-surgery group). For the SR group, second or third staged stent interventions were performed. The study assessed distal remodeling of aortic dissection between the groups, focusing on endpoints such as mortality (both general and aortic-specific), occurrences of visceral branch occlusion, necessity for further interventions, and significant adverse events. Morphological changes were analyzed to determine the therapeutic impact.

**Results:**

The study encompassed 54 participants, with 33 in the SR group and 21 in the non-surgical control group. Baseline demographics and clinical characteristics were statistically comparable across both groups. During an average follow-up of 31.5 ± 7.0 months, aortic-related mortality was 0% in both groups; all-cause mortality was 3% (one case) and 5% (one case) in the SR and control groups, respectively, with no statistically significant difference noted. In the SR group, a single patient experienced complications, including renal artery thrombosis, leading to diminished blood flow. An increased true lumen (TL) area and a decreased false lumen area at various aortic planes were observed in the SR group compared to the control group.

**Conclusion:**

The staged reintervention strategy for treating RAD is safe and provides promising early results.

## Introduction

In patients with acute and chronic aortic dissection, Thoracic Endovascular Aortic Repair (TEVAR) for type B dissections and total aortic arch replacement via the Frozen Elephant Trunk (FET) technique for type A dissections demonstrate favorable early postoperative outcomes. However, these procedures are associated with a high incidence of distal aortic failure, including risks of requiring distal aortic reintervention, distal stent-graft-induced new entry, or aortic-related death. Specifically, patients with more severe and extensive dissections, or those with larger descending aortic diameters as indicated in preoperative computed tomography angiography (CTA), are at a significantly increased risk of developing distal aortic failure (1, 2). The management of residual aortic dissection remains controversial, with long-term aorta-related complications occurring in 20%–50% of patients (3). Moreover, the risk of rupture escalates to approximately 30% once the aortic diameter reaches 60 mm (4). TEVAR has become the preferred treatment for distal dissection due to its minimally invasive approach, supplanting open surgery (5, 6).

For chronic aortic dissection, intervention is recommended when the maximum diameter of the dissection area exceeds 5.5 cm or if there is an increase in aortic diameter of 1 cm annually. Prompt intervention is also crucial for dissections complicated by rupture, malperfusion, intractable pain, or visceral ischemia to prevent further complications (7).

Several risk factors have been identified as influencing the progression toward a descending aorta aneurysm after aortic surgical correction. These include a younger patient age, male gender, a bicuspid aortic valve morphology, a connective tissue disorder, an aortic diameter exceeding 40 mm, false lumen (FL) diameters exceeding 22 mm, and FL patency indicating continuous perfusion within the dissection cavity (8–12). These factors can help identify patients who may benefit from early endovascular intervention to enhance their long-term survival (13, 14).

Several surgical techniques have been proposed for redo surgery after type A or B repair, including endovascular aortic repair with branched and fenestrated endoprostheses. These techniques aim to expand the TL, seal the entry tear, and maintain perfusion to the distal organs (15, 16). However, the complexity and prolonged duration of these procedures pose significant challenges, limiting their broader application in clinical settings. At our center, we have developed a method for addressing distal dissection of the abdominal aorta by dividing it into four zones. Stent placement is tailored to the specific involvement in each zone, facilitating staged surgery. This approach covers entry tears to ensure perfusion to the visceral branches of the abdominal aorta while enlarging the TL. This surgical method provides convenience, high safety levels, and satisfactory clinical efficacy, obviating the need for bespoke temporary stents.

The aim of this study was to analyze the short- to mid-term outcomes of RAD after type A or B aortic repair and to evaluate the effectiveness of staged reintervention for its treatment at a single center.

## Methods

This study received approval from the Institutional Ethics Review Board of the University of Hong Kong-Shenzhen Hospital (Number:hkuszh2023080, 24.05.2023). Patient consent was waived due to the retrospective nature of the study.

### Study population

Patients at risk for poor remodeling of residual aortic dissection post-initial surgery were recruited from our hospital between July 2019 and December 2021. We collected demographic characteristics, as well as preoperative, intraoperative, and postoperative variables.

### Endpoints and morphological analysis

**Figure 1:**
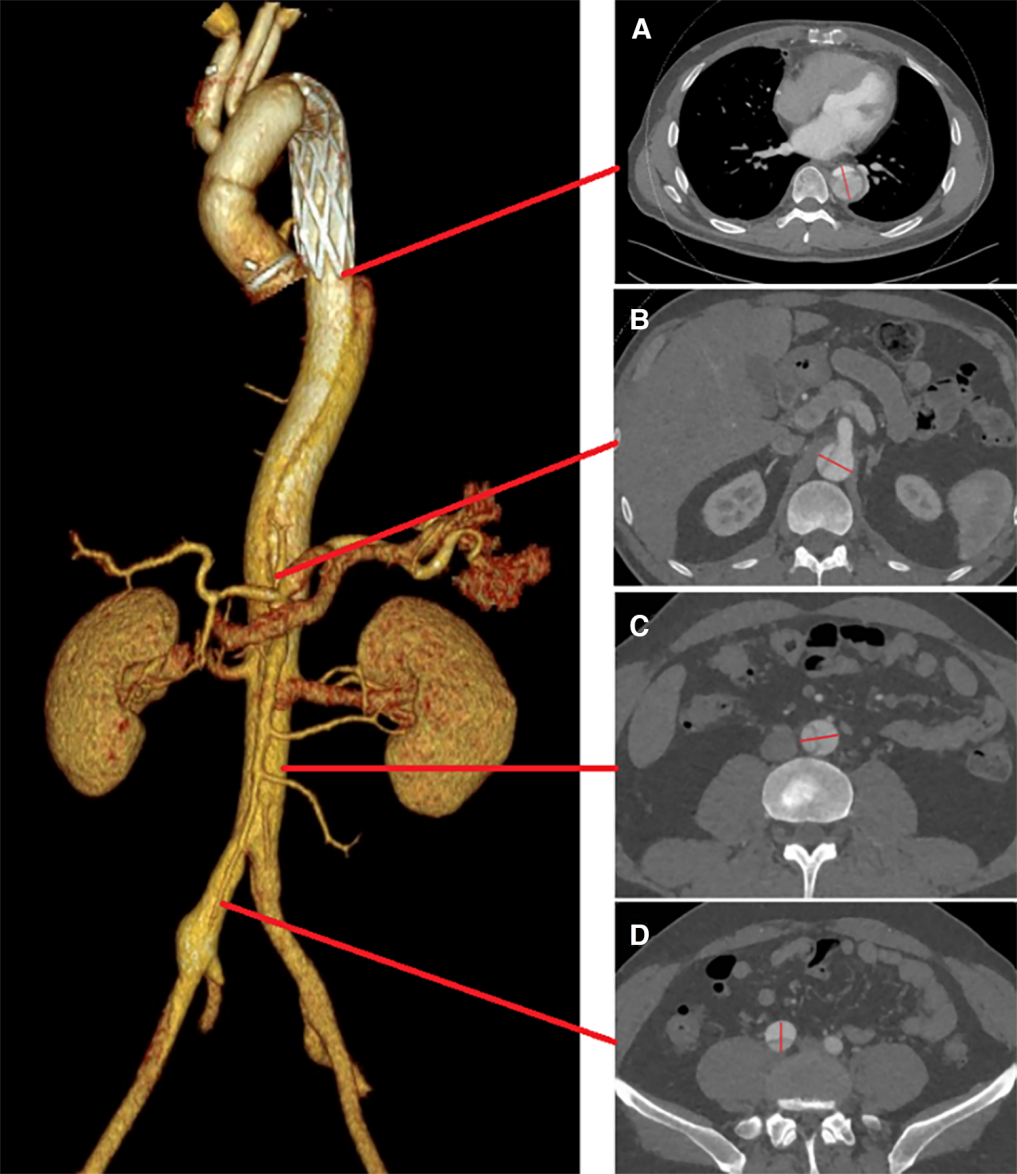
True and FL diameters were measured using a straight line that passes through the midpoint of the intima and the center of the circle. Measurements were taken at the following locations: (**A**) the distal end of the primary stent; (**B**) the celiac trunk opening; (**C**) 5 cm below the renal arteries; (**D**) 1 cm below the iliac artery bifurcation.

The primary outcome of interest was the incidence of all-cause mortality in patients with RAD during the follow-up period. Secondary outcomes included events such as occlusions of visceral branches, the necessity for additional interventions, and incidents categorized as severe adverse events. A comprehensive preoperative morphological assessment was performed, along with regular evaluations at follow-up intervals. This involved precise measurements of TL and FL diameters. Morphological indices were measured at four anatomical planes: the distal end of the primary stent, the plane at the celiac trunk opening, 5 cm below the renal arteries, and 1 cm below the iliac artery bifurcation (Figure 1).

### Inclusion and exclusion criteria

The inclusion criteria were as follows: (1) patients who received initial treatment at our center for a type A or B dissection with RAD; (2) patients presenting with abdominal aortic branch malperfusion; (3) patients with definite entry tears and incomplete thrombosis of the FL in the RAD.

The exclusion criteria were as follows: (1) life expectancy <12 months; (2) ruptured aorta; (3) connective tissue disorder; (4) allergy to aspirin, clopidogrel, contrast agents, anesthetic, or alloy materials; (5) patients lacking postoperative computed tomography scans or follow-up at our center; (6) patients with aortic rupture, intestinal necrosis, or lower limb vascular occlusion.

Initially, 60 patients with residual distal aortic dissection were considered for inclusion. Four patients were excluded due to lack of follow-up and imaging data, one due to postoperative intestinal necrosis, and another due to aortic rupture post-surgery. Ultimately, a total of 54 patients were included in the study.

## Surgical procedures

### Initial surgery for aortic dissection

At our institution, the management of acute type A aortic dissection (ATAAD) began with surgery involving cardiopulmonary bypass (CPB) and circulatory arrest (CA), utilizing moderate hypothermia supplemented by anterograde cerebral perfusion when feasible, or alternatively, employing deep hypothermic conditions. An expeditious total arch replacement (TAR) augmented by the insertion of a frozen elephant trunk (FET) was undertaken for ATAAD patients. For type B aortic dissection, the therapeutic strategy involved TEVAR.

### Residual aortic dissection reintervention

**Figure 2:**
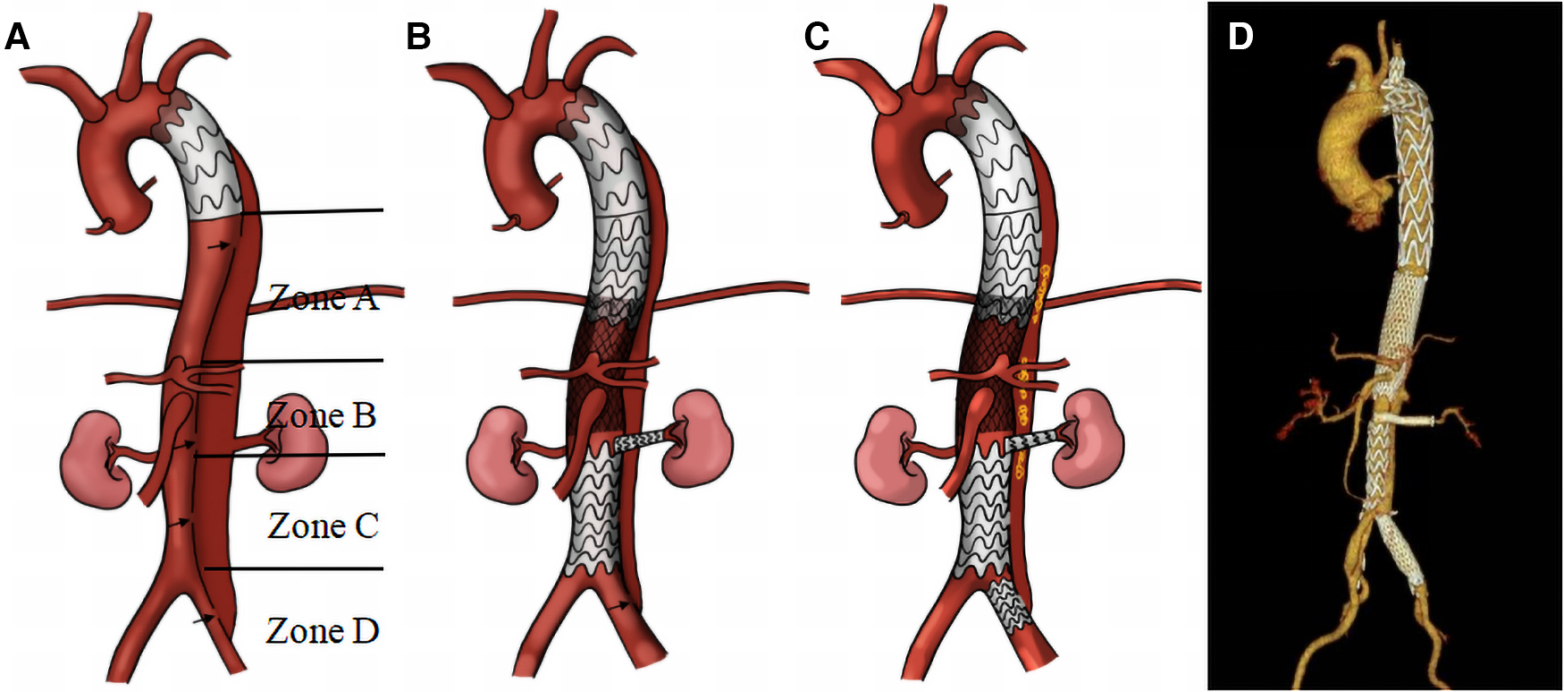
Illustration of the staging reintervention for the remodeling of residual aortic dissection: residual dissection entry tear following the first-stage operation (**A**); second-stage and third-stage operations respectively (**B, C**). This is accompanied by a three-dimensional reconstruction of the thoracic and abdominal aorta following stent implantation (**D**) Black arrow: entry tear; yellow: coil.

Sub-regions: Following initial surgery, the vessels associated with residual aortic dissection in the distal thoracoabdominal aorta were divided into four zones (Zones A–D) (Figure 2).

Zone A: This zone spans from the distal end of the primary stent to above the celiac trunk. In patients experiencing TL compression and entry tears within this area, we implanted straight stent grafts (Microport Medical, Shanghai, China) to expand the TL and sealed the entry tears.

Zone B: This area extends from the celiac trunk to the distal opening of the bilateral renal arteries, referred to as the visceral vascular area of the abdomen. Initially, a self-expandable Bare Stent (Sinus-XL, OptiMed, Germany) was deployed in the visceral aorta to expand its TL. The bare stents, noted for their small pore size, have been crucial in expanding the abdominal aortic TL and promoting thrombosis of the FL.

In situations where the origin of a visceral branch arises from a FL or a significant entry tear is near the branch opening, we strategically positioned a Viabahn stent (Gore, USA) within these branch vessels. One end of the stent is placed in the TL, and the other end extends through the FL into the visceral vascular. The diameter of the Viabahn stent is selected based on the diameter of the adjacent normal artery, with an oversize of 5%–10%, and the length is adjusted to ensure that 3–5 mm remains in the TL of the aorta. This technique not only bridges the true and visceral vascular lumens but also effectively seals the entry tear adjacent to the viscera vascular.

Zone C: This zone encompasses the region from the distal end of the opening of the bilateral renal arteries to above the iliac artery bifurcation. In patients with compression and entry tears in this are, straight stent grafts were employed to expand the TL and seal entry tears.

Zone D: This area refers to the region below the iliac artery bifurcation. If there is a re-entry tear in this area, blood would flow back through the iliac artery entry tear into the abdominal aorta, resulting in incomplete thrombosis of the FL. To promote thrombosis of the FL, coils (Interlock, Boston, USA) were placed from the iliac artery re-entry to the FL plane of the abdominal aorta. Subsequently, a Fluency Plus stent graft (Bard, USA) was used to seal the iliac artery entry tear.

Multi-stage interventions: Based on the results of aortic Computed Tomography Angiography (CTA), patients who have undergone the initial stage of the operation will proceed with second-stage or third-stage stent implantations.

Second-stage surgery: Approximately one month after the initial surgery, aortic CTA was reviewed. If necessary, lesions in Zones A, B, and C were treated first. In cases with lesions across different zones, various stent implantation methods were used to seal entry tears and expand the TL of the residual aortic dissection. Three primary methods were utilized for the second-stage surgery. The first method involved using a straight stent graft (Microport Medical, Shanghai, China) for patients with a distinct entry tear between the true and FL in Zones A and C, effectively sealing the entry tear. The second method used the Sinus-XL bare stent (OptiMed, Germany) for patients with TL narrowing in the visceral vessel area (Zone B). This stent, chosen to expand the TL and decelerate FL flow to promote thrombosis, was used in conjunction with balloon dilation to improve the apposition between the intimal and adventitial layers of the dissection, thus reducing the FL volume. This approach is consistent with the “provisional extension to induce complete attachment technique (PETTICOAT technique)” and “stent-assisted balloon-induced intimal disruption and relamination of aortic dissection (STABILISE technique)” (17, 18). The third method involved using a Viabahn stent (Gore, USA) in visceral arteries for patients with a distinct entry tear near visceral branch vessels, placing the stent to seal the entry tear. The average duration of the second-stage surgery was 44 ± 18 min.

**Figure 3:**
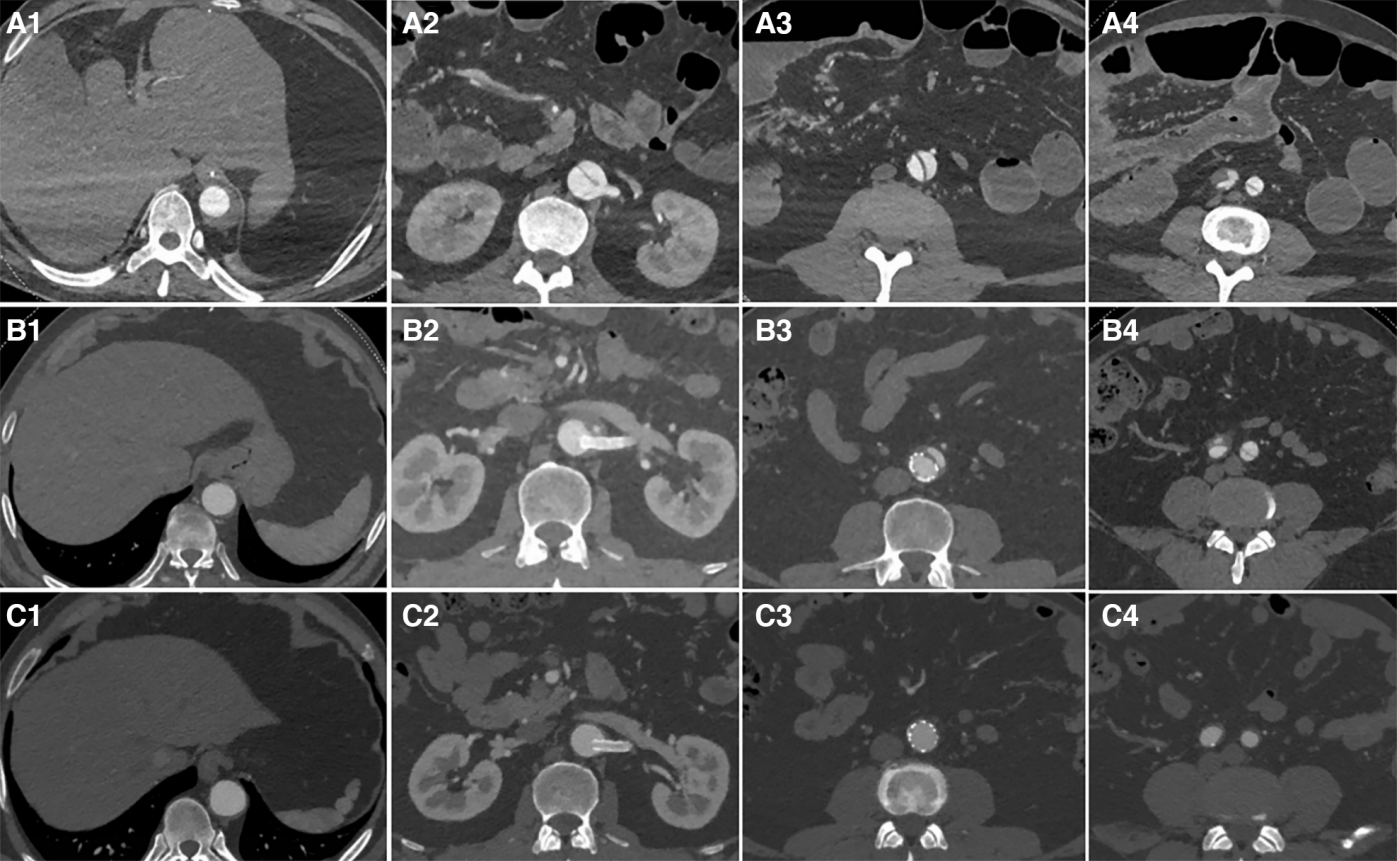
The first row displays preoperative CT images, the second row presents CT images taken three months after the second-stage surgery, and the third row features follow-up CT images taken one year after the second-stage surgery. From left to right, the images respectively show sections of aortic dissection at the distal end plane of the primary stent, the renal artery level, 5 cm below the renal artery level, and 1 cm below the bifurcation of the iliac artery. After initial surgery, zones (**A–D**) exhibited a residual FL that had not fully thrombosed (A1-A4). Three months after the second-stage surgery, the aortic CTA revealed TL re-expansion without any obvious entry tears; follow-up observation was subsequently continued (B1-B4). One year after second-stage surgery, aortic CTA revealed that the FL had completely thrombosed and disappeared (C1-C4).

Third-stage surgery: Approximately three months after the second-stage surgery, aortic CTA was re-examined. Patients with thrombosis of the FL and no significant entry tear were followed up for an additional year (Figure 3). In cases where the FL was not completely thrombosed, often due to an entry tear in the iliac artery causing blood to flow back into the FL of the abdominal aorta, coils were placed from the iliac artery entry tear into the FL to promote thrombosis. Subsequently, a Fluency Plus stent graft was placed in the iliac artery to seal the re-entry. The average duration of the third-stage surgery was 32 ± 5 min.

## Surgical methods

All patients received local anesthesia with monitored anesthesia care. In cases where patients were uncooperative, general anesthesia was administered.

The modified Seldinger technique was employed, involving the puncture of the femoral artery on one side (preferably the side adjacent to the aortic lumen) followed by the insertion of a 5F arterial sheath. Initially, two Perclose ProGlide vascular sutures were pre-embedded. Subsequently, a 14–20F femoral artery sheath was implanted, sized according to the diameter of the aortic stent. Guidewires facilitated the insertion of pigtail catheters for angiography of the abdominal, descending, and ascending aorta. These procedures aimed to ascertain the diameter of the proximal anchoring zone of the dissection, the location and size of the entry tear, and its relationship with the branches of the abdominal aorta. The angiographic findings guided the selection of the appropriate aortic stent size. Due to inadequate dilation in the thoracic and abdominal aortic segments, the TL was initially expanded from Zones A to C, covering multiple small entry tears in Zones A or C. Under guidewire supervision, stents (straight stent grafts, Sinus-XL bare stents) were strategically placed in various aortic sections as required. Repeat angiography of the abdominal aorta was conducted to verify the position of the entry tear at the abdominal aortic branch, assess the flow rate and velocity of the contrast medium into the FL, measure the width of the FL and the diameter of the involved abdominal branch arteries, and conclude with the placement of stents in the abdominal branch arteries.

During angiography of the abdominal aortic branches, it is essential to perform both intramural tangential imaging and selective imaging of the involved arteries, which should be coordinated with a pathway map for stent implantation. A flexible long sheath, used in conjunction with a hydrophilic guidewire, enables access to branch vessels. Under guidewire guidance, Viabahn stents are implanted in the abdominal branch vessels. Diameter selection is based on the diameter of the adjacent normal artery, with an oversizing of 5%–10%, and the length must ensure that 3–5 mm of the aortic lumen is preserved. If the flow rate in the FL remains high after stent implantation, this issue may be addressed further by using coils to occlude the FL.

Patients with stent placement in the visceral branches of the abdominal aorta routinely received dual antiplatelet therapy (aspirin 100 mg plus clopidogrel 75 mg once daily), unless contraindicated. Patients without stent placement in the abdominal aorta branches received standard antiplatelet treatment with either aspirin 100 mg or clopidogrel 75 mg once daily, along with acid suppression therapy using pantoprazole. Technical success was defined as the absence of a “type I” endoleak, no conversion to open surgery, and no mortality within 24 h post-surgery.

### Statistical analysis

In the assessment of categorical variables, discrepancies were identified using the chi-square test or Fisher's exact test, depending on their applicability. For continuous variables, the Student's *t*-test was used to compare means for variables with normal distributions, while nonparametric tests were employed for data that deviated from normality. The Shapiro-Wilk test was applied to assess the normality of variable distributions. Continuous variables are presented as mean ± standard deviation, and categorical variables are shown as frequencies and proportions.

Survival rates over time were calculated using the Kaplan-Meier estimator. Bidirectional statistical testing was conducted, with significance attributed to *p*-values less than 0.05. Analytical procedures were conducted using IBM SPSS Statistics 25.0 (IBM Corp, Armonk, NY) and GraphPad Prism software (version 9.5; GraphPad Software, San Diego, CA).

## Results

### Patient cohort

Between July 2019 and December 2021, a total of 54 patients were included in the study (Table 1), comprising 50 males (50 /54, 93%) and 4 females (4/54, 7%), aged from 21 to 79 years, with an average age of 46.0 ± 10.8 years. The study consisted of 33 patients in the SR group and 21 in the control group. Preoperative radiological data are presented in Table 2.

**Table 1:** Baseline characteristics of the 54 patients in the study.

Variables	SR	Control	*P*-value
Total	33	21	
Male	31 (94%)	19 (90%)	0.636
Age (years)	44.8 ± 9.9	48.0 ± 12.2	0.293
Weight (kg)	74.8 ± 8.2	77.7 ± 12.2	0.354
Height (cm)	170.5 ± 5.5	170.1 ± 6.5	0.829
TAAD after open	15 (45%)	13 (62%)	0.238
TBAD after TEVAR	18 (55%)	8 (38%)	0.238
Past medical history			
Hypertension	25 (76%)	12 (57%)	0.151
Hyperlipidemia	4 (12%)	3 (14%)	0.817
Diabetes mellitus	3 (9%)	2 (10%)	0.957
Coronary artery disease	1 (3%)	1 (5%)	0.743
Cerebral infarction	1 (3%)	0 (0%)	0.421
Renal transplant	1 (3%)	1 (5%)	0.743
Personal History			
Smoking	6 (18%)	5 (24%)	0.617
Alcohol use	3 (9%)	2 (10%)	0.957

SR, staging reintervention; TAAD, type A aortic dissection; TBAD, type B aortic dissection; TEVAR, Thoracic endovascular aortic repair.

**Table 2:** Clinical and radiological data of 54 patients in the study.

Variables	SR	Control	*P*-value
Involvement of distal aortic dissection			
Above celiac artery plane	2 (6%)	0 (0%)	0.25
Below celiac artery plane	31 (94%)	21 (100%)	0.25
Pattern of perfusion in visceral arteries			
Celiac trunk			
Arising from the true lumen	21 (64%)	11 (52%)	0.412
Arising from the false lumen	1 (3%)	7 (33%)	0.002
Arising from the mixed lumen	11 (33%)	3 (14%)	0.119
Superior mesenteric artery			
Arising from the true lumen	25 (76%)	16 (76%)	0.971
Arising from the false lumen	0	0	
Arising from the mixed lumen	8 (24%)	5 (24%)	0.971
Renal artery			
Arising from the true lumen	12 (36%)	5 (24%)	0.333
Arising from the false lumen	16 (48%)	12 (57%)	0.535
Arising from the mixed lumen	5 (15%)	4 (19%)	0.708

SR, staging reintervention.

### Intraoperative stent placement

In the SR group, 15 patients with Stanford type A dissection and 18 with type B dissection were enrolled. A total of 25 stents were placed in the abdominal aorta visceral branches: 23 patients received stent insertion in Zone A, 16 in Zone B, 23 in Zone C, and 16 in Zone D (Table 3). During the procedures, a few complications occurred. One patient experienced thrombosis and reduced blood flow in the distal renal artery following the placement of a renal artery stent. This patient developed low back pain, which gradually improved with anticoagulant therapy.

**Table 3:** Stent placement at different sites during second- and third-stage surgery in SR group.

Dissection	N	Zone A (straight stent)	Zone B					Zone C (straight stent)	Zone D FVL + coil
			(Sinus-XL)	Stents in abdominal aorta branches (VB stent)					
				CT	SMA	Left RA	Right RA		
Type A	15	10	7	0	2	6	3	11	8
Type B	18	13	9	2	1	6	5	12	8
Total	33	23	16	2	3	12	8	23	16

SMA, superior mesenteric artery; RA, renal artery; CT, celiac trunk; FVL, fluency plus stent graft; VB, viabahn stent.

### Follow-up analysis

**Figure 4:**
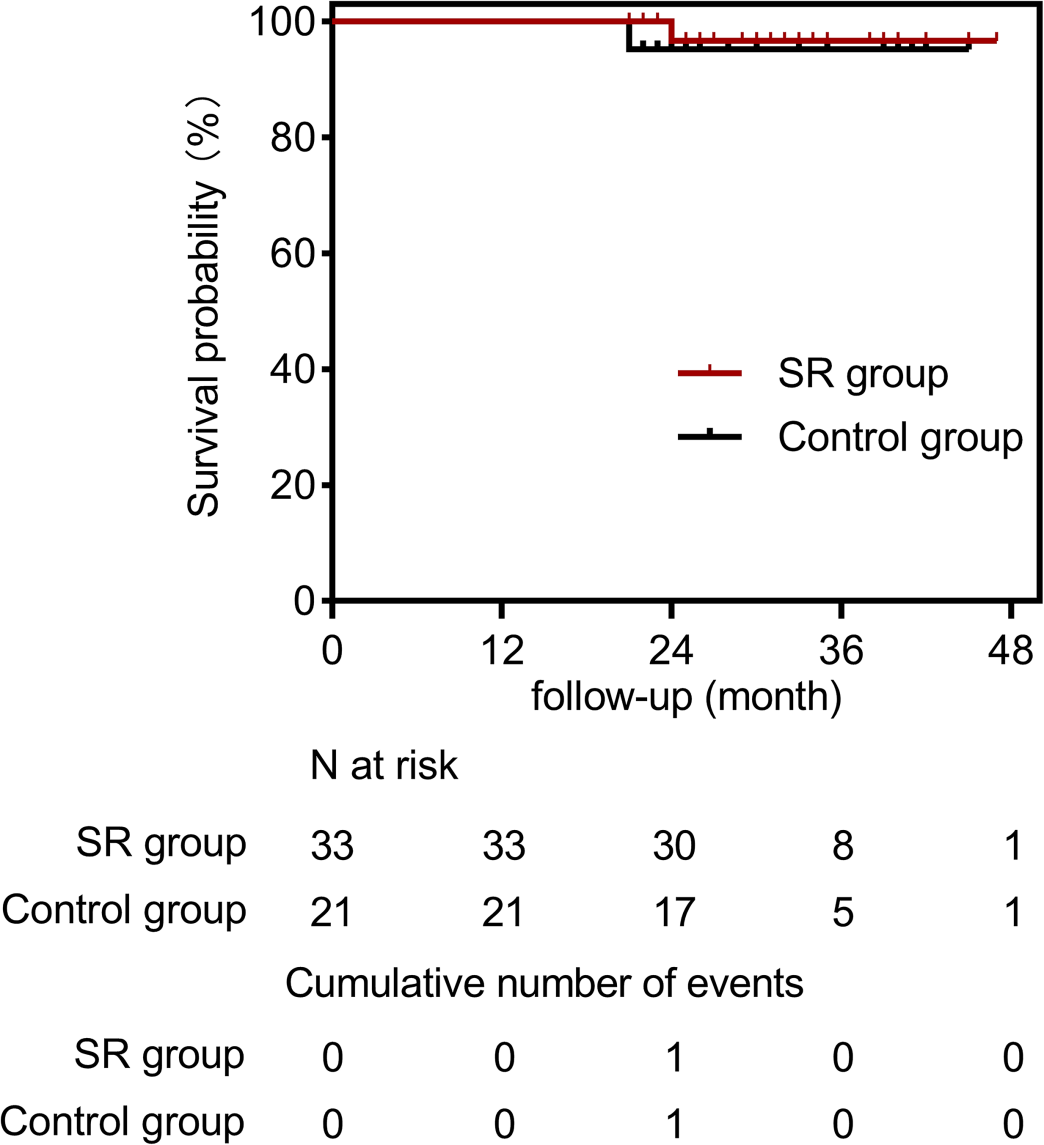
Cumulative kaplan-meier estimate for freedom from all-cause mortality in the SR group (solid red line) and the control group (solid black line).

The mean follow-up duration was 31.5 ± 7.0 months (range, 21–47 months). We compared the follow-up results of the two groups in Table 4. In the SR group, one patient died during the follow-up period due to multiple organ dysfunction caused by COVID-19. In the control group, one patient was diagnosed with a heart tumor during follow-up and unfortunately died from heart rupture. Two patients were lost to follow-up at 21 and 30 months, respectively (Figure 4). Aortic CTA examinations revealed favorable stent morphology, with no evidence of stent migration, reduced FL, or expanded TL. None of the patients experienced gastrointestinal bleeding. In one case, a switch from clopidogrel to ticagrelor was necessary due to an allergy to clopidogrel. A patient experienced thrombosis at the distal end of one renal artery during surgery. However, during follow-up, there were no symptoms of discomfort such as low back pain, and the patient's creatinine levels remained normal, despite reduced blood flow in the renal artery compared to preoperative levels.

**Table 4:** Follow-up results.

Variables	SR	Control	*P*-value
No.	33	21	
Follow-up			
Mean follow-up (months)	32.0 ± 6.9	30.7 ± 7.3	0.492
Drop out	1 (3%)	1 (5%)	0.743
Clinical outcomes			
All-cause mortality	1 (3%)	1 (5%)	0.743
Stent-related mortality	0%	0%	
Branch vessels occluded	1 (3%)	0 (0%)	0.421
Severe adverse events			
Retrograde TAAD	0	0	
Paraplegia	0	0	
Hemorrhage	0	0	
Acute kidney injury	1 (3%)	0 (0%)	0.421

### RAD evolution in SR group

**Figure 5:**
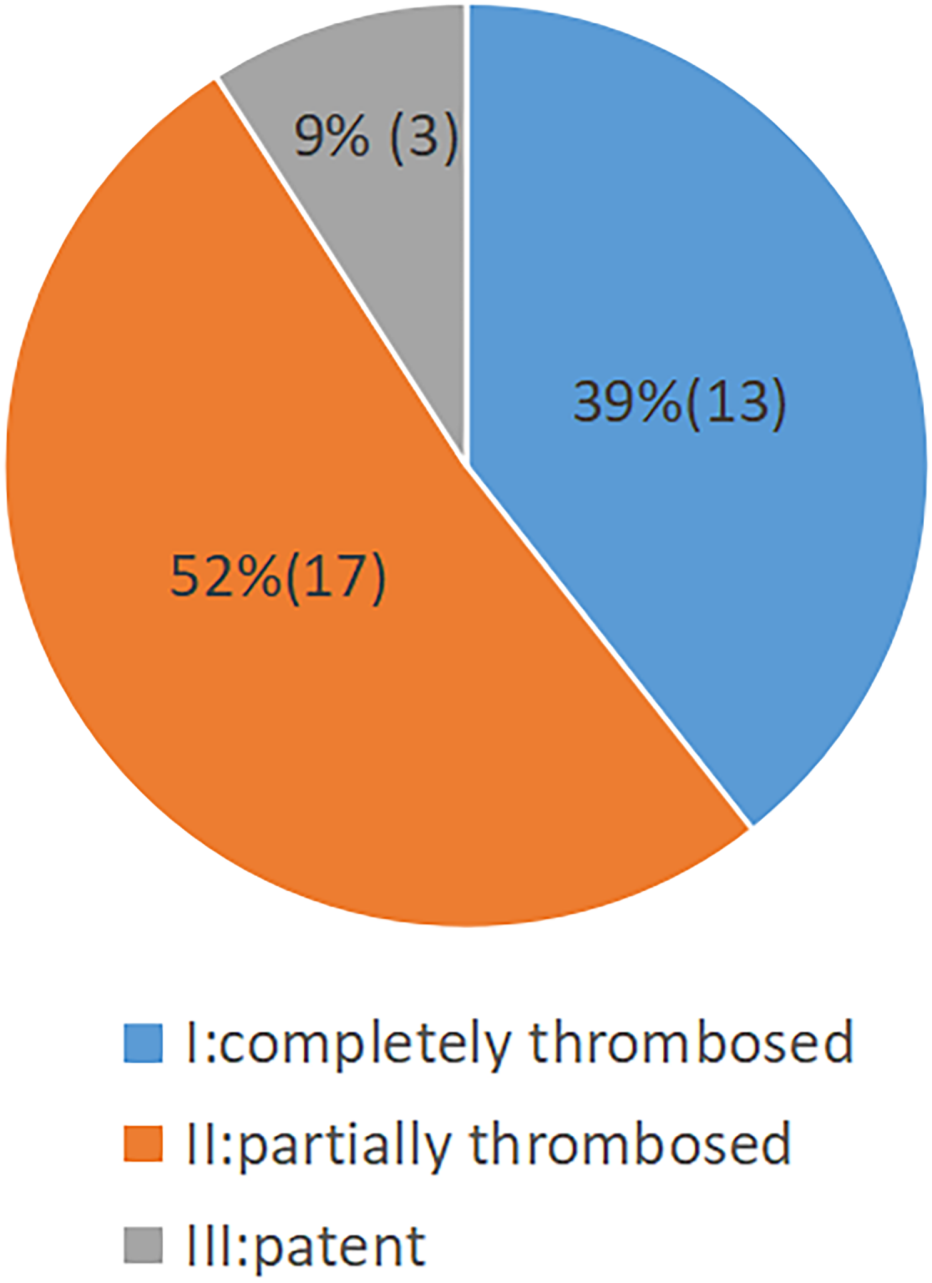
Pie chart showing the status of aortic FL thrombosis following staging reintervention (*n* = 33).

**Figure 6:**
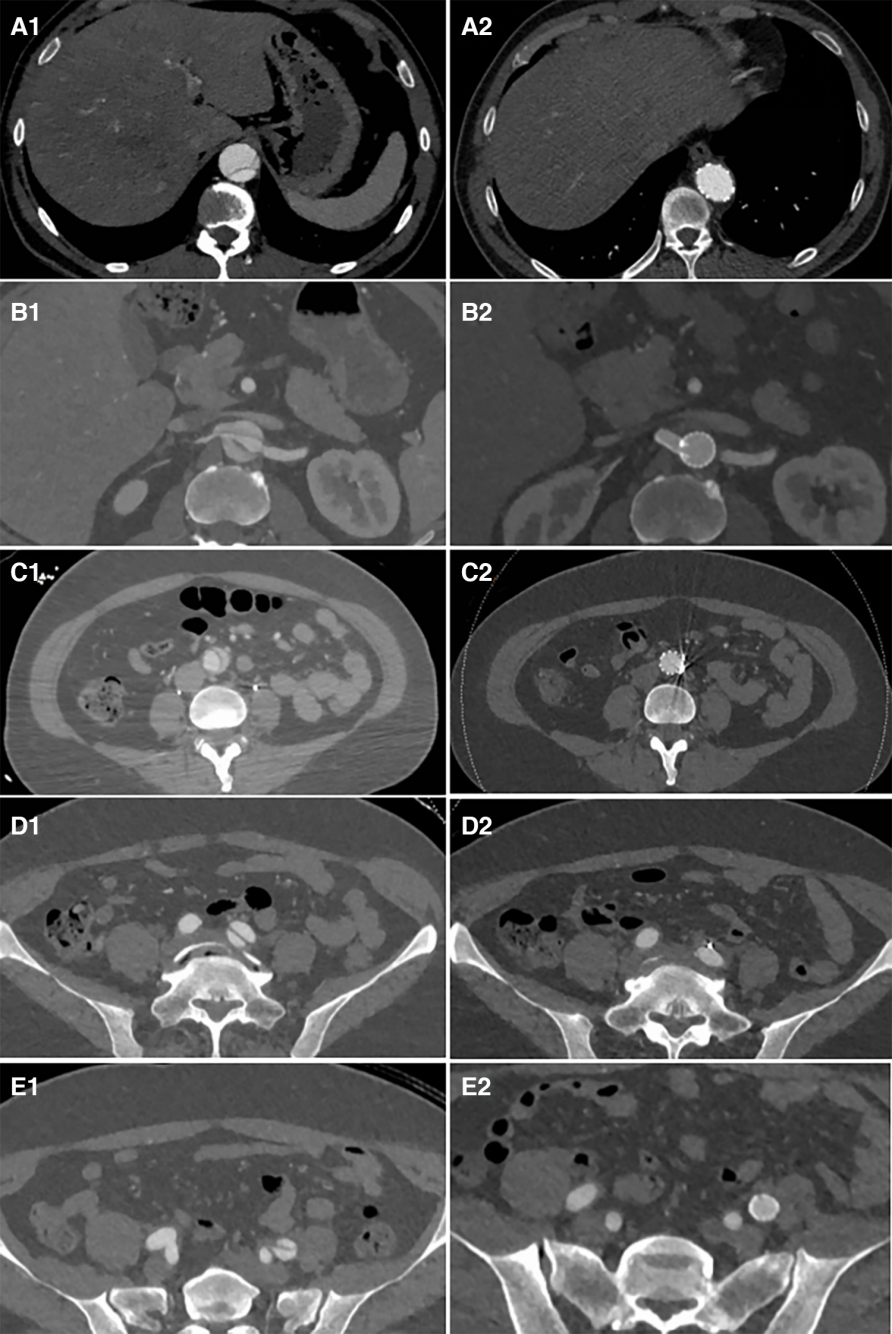
Distal plane of the stent during initial surgery: the TL was narrowed and underwent significant expansion after the second-stage surgery (A1 and A2). The plane of the renal artery opening: After stents were placed in the right renal artery and abdominal artery, the TL of the visceral branch area expanded, and the FL completely disappeared (B1 and B2). The narrowest plane of the TL of the abdominal aorta below the renal artery: Both the TL expanded and the FL disappeared after stent placement (C1 and C2). After the stent placement in the external iliac artery to cover the iliac artery entry tear, the FL disappeared (D1, D2, E1, and E2).

The status of the residual aortic dissection FL was classified by imaging as completely thrombosed or disappeared (type I), partially thrombosed (type II), or patent (type III). The rate of complete thrombosis or disappearance was 39% (13/33); the rate of partial thrombosis was 52% (17/33); the rate of patent FL was 9% (3/33) (Figure 5). A representative case of aortic remodeling is presented in Figure 6.

### Aortic remodeling

In the SR group, a significant reduction in the FL diameter with a corresponding increase in the true lumen diameter was observed after stent-graft placement (*p* < 0.001). There was no significant change in the TL diameter between preoperation and follow-up in the control group. However, the FL showed progressive dilation during the follow-up period (*P* < 0.001).

**Figure 7:**
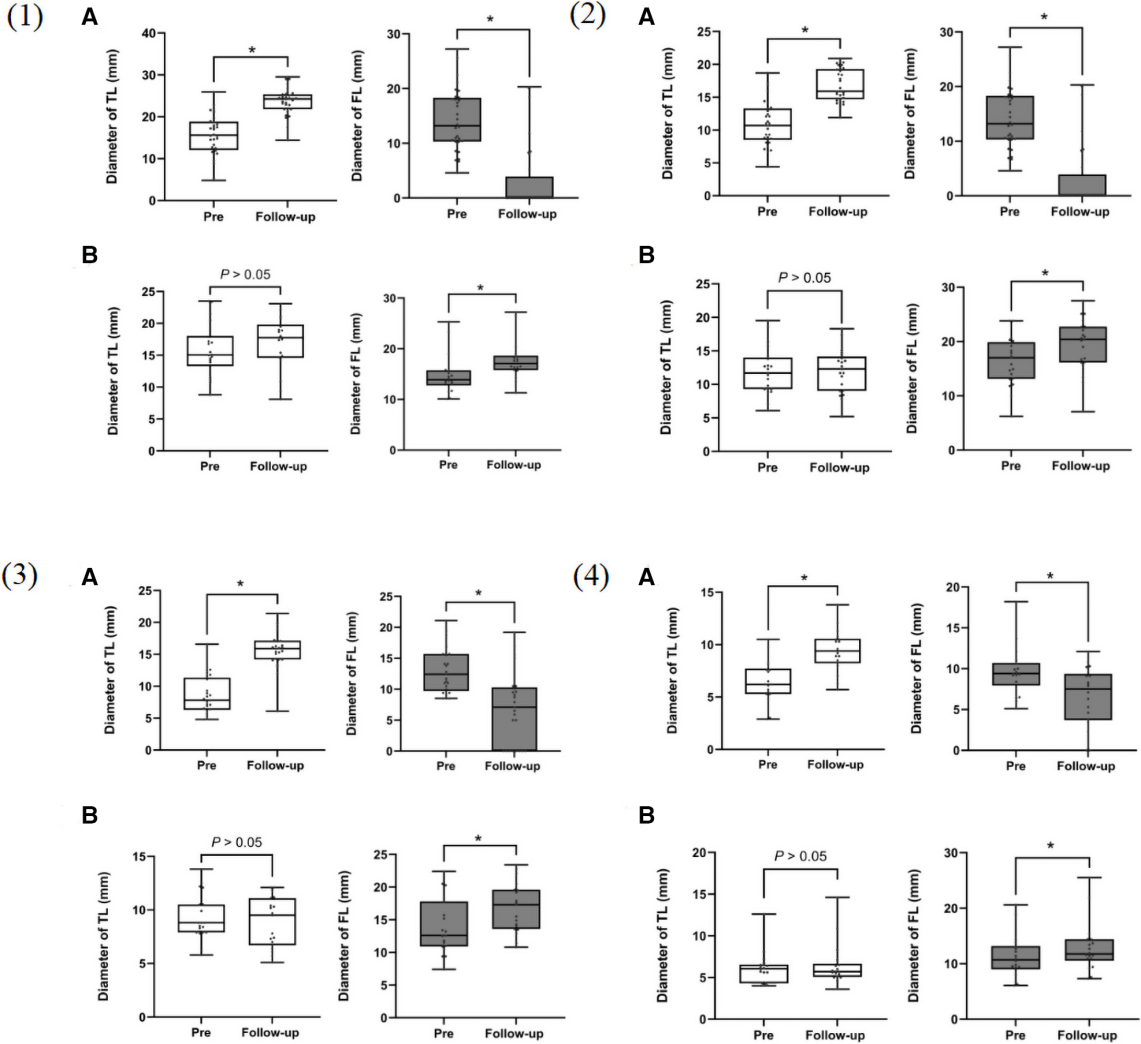
Box plots for aortic measurements from 54 patients using actual measurement times before surgery and at the last follow-up computed tomography angiography (CTA). (**A**) staging reintervention (SR) group; (**B**) control group. (1): at the distal end plane of the primary stent; (2): at the plane of the celiac trunk opening; (3): 5 cm below the renal arteries; (4): 1 cm below the iliac artery bifurcation. TL, true lumen; FL, false lumen. *p < 0.001.

Most cases in the SR group demonstrated favorable aortic remodeling in the stent-covered area, with an expansion of the TL diameter and a decrease in the FL diameter during the follow-up period (Figure 7 and Table 5).

**Table 5:** Status of aortic lumen and size in follow-up.

Group	Variable	True lumen (mm)			False lumen (mm)		
		Pre	Follow-up	*P*	Pre	Follow-up	*P*
SR	Zone A	15.6 ± 5.0	23.7 ± 3.2	<0.001	14.2 ± 5.7	3.2 ± 6.3	<0.001
	Zone B	11.0 ± 3.4	16.8 ± 2.5	<0.001	13.7 ± 4.8	7.0 ± 6.8	<0.001
	Zone C	8.5 ± 3.0	15.6 ± 3.0	<0.001	13.1 ± 3.7	6.9 ± 5.9	<0.001
	Zone D	6.5 ± 2.0	9.4 ± 2.0	<0.001	9.7 ± 3.0	6.6 ± 3.6	<0.001
Control	Zone A	15.8 ± 4.2	16.9 ± 4.3	0.163	14.9 ± 4.0	17.7 ± 3.9	<0.001
	Zone B	12.0 ± 3.5	12.0 ± 3.3	0.959	16.4 ± 4.5	19.3 ± 4.9	<0.001
	Zone C	9.3 ± 2.2	8.8 ± 2.4	0.115	13.9 ± 4.2	16.9 ± 3.8	<0.001
	Zone D	6.1 ± 2.0	6.5 ± 2.6	0.426	11.3 ± 3.9	13.0 ± 4.6	<0.001

## Discussion

Initial surgery for proximal aortic dissection is crucial in preserving blood flow to the branches of the aortic arch. However, the timely management of residual aortic dissection continues to be debated, especially when patients lack specific symptoms and laboratory tests fail to show dysfunction in abdominal organs. Previous studies have indicated that more than 30% of cases with residual aortic dissection undergo continuous enlargement of the FL, resulting in increased medium- and long-term mortality (19). The presence of a distal entry tear further elevates the risk of late aortic rupture and hinders aortic remodeling (20). Gasparetto et al. reported that 58.3% of patients with two or more entry tears in the distal aorta exhibited enlargement of the FL (>5 mm) during an average follow-up of 27 months (21). These findings suggest that residual entry tears adversely affect postoperative aortic remodeling and contribute to persistent non-healing of the FL. Moreover, studies have demonstrated a direct association between FL patency and distal aneurysmal dilatation (22). Therefore, timely intervention can enhance aortic remodeling in patients with residual aortic dissection characterized by FL patency, TL compression, and incomplete thrombosis of the FL.

Endovascular repair has become the preferred method for treating Stanford type B dissection due to its minimally invasive nature and reduced complication rate compared to open surgery. However, reconstructing the visceral branches of the abdominal aorta poses a significant challenge (23). Various techniques, including open surgery, branch stent technology, and the fenestration technique, have been employed for abdominal branch reconstruction (24–26). The fenestration technique enables the preservation of visceral branch perfusion by creating a hole through which a covered stent can be inserted to expand the TL. Although this procedure is highly technical and demands precision, branch stent implantation effectively sustains the perfusion of visceral branches in patients with aortic aneurysms, albeit challenging in cases with TL compression (27, 28). These surgical methods are not only complex and demanding but also associated with high postoperative complication and mortality rates, necessitating specialized vascular centers and experienced surgeons (29, 30). In contrast, the staged approach used at our center is comparatively simpler and can be customized to individual patient conditions.

At our center, we have encountered cases where patients presented with entry tears below the renal artery. In these instances, a covered stent was placed below the renal artery to seal the entry tear and expand the TL. This approach, which involves covering the inferior mesenteric artery without causing intestinal ischemia, suggests that releasing the stent below the renal artery and covering the inferior mesenteric artery is safe provided the superior mesenteric artery remains unobstructed.

Studies have demonstrated that increased coverage of the thoracic aorta with stent grafts can elevate the risk of spinal cord ischemia. The reported incidence of symptomatic spinal cord ischemia following TEVAR ranges between 1% and 5% (31). Furthermore, patients considered at high risk are those who require TEVAR with extensive coverage of the thoracic aorta (32). In our approach, we position endografts to cover the entry tear and promote FL thrombosis for patients with TL compression below the renal artery and above the celiac trunk, as well as those with large or multiple entry tears. Importantly, during postoperative follow-up, we observed no cases of paraplegia. Staged repair also allows adequate time for the development of collateral circulation in the spinal cord, potentially reducing the risk of paraplegia. Notably, none of our cases developed paraplegia as a complication of the procedure.

Currently, no expert consensus or guideline recommends the optimal timing for endovascular aortic repair in the treatment of residual aortic dissection. Based on our clinical experience, we have determined that performing the second-stage operation approximately one month after the initial surgery produces favorable outcomes. This timing facilitates the softening and easy expansion of the dissection intima, establishes collateral circulation in the abdominal aorta, and lowers the risk of postoperative paraplegia. Three months after the second-stage operation, a reexamination was performed to assess progress. In the third stage, coils were used to promote thrombosis of the FL in patients who did not exhibit complete thrombosis, and stents were employed to cover the re-entry in the iliac artery. The staging approach adopted by our center enables personalized stent placement and avoids a “one-size-fits-all” situation. Our approach is less invasive, simpler to perform, and associated with shorter operation times compared to more complex methods for treating residual aortic dissection. Our study demonstrated that this method effectively seals the dissection entry tear, promotes FL thrombosis, and improves the remodeling of residual aortic dissection.

This study is limited by its comparison with historical data; the short- to mid-term follow-up duration limits our ability to assess the long-term effects of this technique. Additionally, as a single-center retrospective study, it is susceptible to selection bias, and therefore, a larger-scale, prospective, randomized trial is necessary to confirm these findings.

## Conclusion

Staging stent placement as a viable and relatively simpler approach can effectively improve the remodeling of the distal aortic dissection. By targeting different aorta zones based on the extent of dissection involvement, stent intervention can be effectively implemented. The short- to mid-term outcomes of this surgical method have demonstrated satisfactory efficacy. However, to confirm the long-term efficacy, further follow-up studies are necessary.

## Data Availability

The original contributions presented in the study are included in the article/Supplementary Material, further inquiries can be directed to the corresponding authors.
